# Shifting from Population-wide to Personalized Cancer Prognosis with Microarrays

**DOI:** 10.1371/journal.pone.0029534

**Published:** 2012-01-25

**Authors:** Li Shao, Xiaohui Fan, Ningtao Cheng, Leihong Wu, Haoshu Xiong, Hong Fang, Don Ding, Leming Shi, Yiyu Cheng, Weida Tong

**Affiliations:** 1 Pharmaceutical Informatics Institute, College of Pharmaceutical Sciences, Zhejiang University, Hangzhou, China; 2 The Wallace H. Coulter Department of Biomedical Engineering, Georgia Institute of Technology and Emory University, Atlanta, Georgia, United States of America; 3 ICF International at NCTR/FDA, Jefferson, Arkansas, United States of America; 4 National Center for Toxicological Research (NCTR), U. S. Food and Drug Administration, Jefferson, Arkansas, United States of America; Michigan State University, United States of America

## Abstract

The era of personalized medicine for cancer therapeutics has taken an important step forward in making accurate prognoses for individual patients with the adoption of high-throughput microarray technology. However, microarray technology in cancer diagnosis or prognosis has been primarily used for the statistical evaluation of patient populations, and thus excludes inter-individual variability and patient-specific predictions. Here we propose a metric called clinical confidence that serves as a measure of prognostic reliability to facilitate the shift from population-wide to personalized cancer prognosis using microarray-based predictive models. The performance of sample-based models predicted with different clinical confidences was evaluated and compared systematically using three large clinical datasets studying the following cancers: breast cancer, multiple myeloma, and neuroblastoma. Survival curves for patients, with different confidences, were also delineated. The results show that the clinical confidence metric separates patients with different prediction accuracies and survival times. Samples with high clinical confidence were likely to have accurate prognoses from predictive models. Moreover, patients with high clinical confidence would be expected to live for a notably longer or shorter time if their prognosis was good or grim based on the models, respectively. We conclude that clinical confidence could serve as a beneficial metric for personalized cancer prognosis prediction utilizing microarrays. Ascribing a confidence level to prognosis with the clinical confidence metric provides the clinician an objective, personalized basis for decisions, such as choosing the severity of the treatment.

## Introduction

Not all individuals respond to drug treatment in the same way. Accordingly, the development of personalized therapeutic regimens optimized for individual patients represents a major goal of 21^st^-century medicine [1]. Modern tools are being utilized to assist physicians in effectively treating patients as individuals and providing personalized drug intervention.

Inter-individual variation in response to drug treatment is strongly influenced by a patient's physiological state at the time of treatment. This state can be characterized by gene expression profiles [2]. Therefore, microarray technology can guide the selection of drugs or therapeutic regimens and be employed to assess the susceptibility of a patient to certain diseases, enabling a personalized plan for prevention monitoring and treatment [3]. The prospective benefits of microarray technology in clinical applications have been demonstrated by several landmark studies [4]–[7]. Microarray-based predictive models (or genomic signatures) have shown utility in associating different subgroups of breast cancer with distinct clinical outcomes [8]–[13], such as MammaPrint™ [4], [5], a milestone in microarray-based prognosis for breast cancer [14].

The development of a microarray-based predictive model for tumor classification typically involves two sequential steps [4], [15]–[17]. First, the model is developed based on a training set of patients with known class labels (e.g., tumor status) and gene expression data. Next, the training model is validated using a validation set that contains patients with known class labels. The validity of the training model in performance on the validation set has been the focus of ‘class prediction’ research. To ensure the training model can be used in real-world clinical applications, it was suggested that the model must be assessed on a large number of independent samples in this external validation process [18].

It is important to note that the aforementioned external validation strategy assesses the performance of a training model using a population defined by the validation set. The average performance (e.g., specificity, sensitivity) over the population is used to assess whether the model can be a reliable diagnostic or prognostic test. This strategy is performed under the assumption that the model performs equally for everyone without considering the inter-individual variability. Thus, the average performance based on a population of patients cannot ensure its predictive ability for individual patients, which might result in potentially unreliable diagnoses or prognoses in the real-world application. This one-size-fits-all strategy needs to be modified from population-wide to personalized medicine in microarray data-based applications.

We propose a metric called clinical confidence that measures the model's reliability in prediction performance on an individual basis. Clinical confidence can be useful in determining appropriate treatments; for example, patients with high confidence and poor prognosis may be given more rigorous treatments. Additionally, patients with lower clinical confidences may be prime candidates for further evaluation of their conditions with alternative methods. The accuracy of the clinical confidence metric was investigated on three large clinical datasets with total of six clinical endpoints [19].

Specifically, we first divided each dataset into two, i.e., the training and validation set. To mimic real-world clinical scenarios, we made the validation set that contains only the patients whose microarray data were generated at a later date than those in the training set. We derived the clinical confidence from the training model, followed by the assessment of its correlation with prediction accuracy for prognosis and the survival time of the patients in the validation set. To the best of our knowledge, this is the first attempt to provide a measure of confidence for individual patients in microarray-based “class prediction” research, which is an important step forward in personalized medicine.

## Materials and Methods

### Datasets

Three large-scale, clinical cancer datasets were used in this study: breast cancer (BR) [20], multiple myeloma (MM) [21], and neuroblastoma (NB) [22]. A concise summary of the datasets is given in **Table 1**. More detailed information of these datasets can be found in the main paper of the second phase of MicroArray Quality Control project (MAQC-II) [19].

**Table 1:** A concise summary of datasets.

Data Set code	Endpoint Description	Endpoint Code*	Sample Size		Ratio of events		Microarray Platform (number of channel)
			Training	Validation	Training	Validation	
BR	Treatment Response	BR-pCR	130	100	0.34 (33/97)†	0.18 (15/85)	Affymetrix U133A (1)
		BR-erpos	130	100	1.60 (80/50)	1.56 (61/39)	
MM	Overall Survival Milestone Outcome	MM-OS	340	214	0.18 (51/289)	0.14 (27/187)	Affymetrix U133Plus2.0 (1)
	Event-free Survival Milestone Outcome	MM-EFS	340	214	0.33 (84/256)	0.19 (34/180)	
NB	Overall Survival Milestone Outcome	NB-OS	246	177	0.32 (59/187)	0.28 (39/138)	Agilent NB Customized Array (2)
	Event-free Survival Milestone Outcome	NB-EFS	246	193	0.65 (97/149)	0.75 (83/110)	
Control	Positive control	NB-PC	246	231	1.44 (145/101)	1.36 (133/98)	Agilent NB Customized Array (2)
		MM-PC	340	214	1.33 (194/146)	1.89 (140/74)	Affymetrix U133Plus2.0 (1)
	Negative control	NB-NC	246	253	1.44 (145/101)	1.30 (143/110)	Agilent NB Customized Array (2)
		MM-NC	340	214	1.43 (200/140)	1.33 (122/92)	Affymetrix U133Plus2.0 (1)

*BR - Breast Cancer; MM - Multiple Myeloma; NB - Neuroblastoma; pCR - Pathologic Complete Response; erpos – ER Positive; OS – Overall Survive; EFS – Event-free Survival; PC – Positive Control; NC – Negative Control.

† Ratio of good to poor prognoses (i.e., good/poor prognoses).

Each dataset has two clinical endpoints related to cancer prognosis (including survival data) or treatment: BR-pCR and BR-erpos in the treatment response dataset, NB-EFS and MM-EFS in the event-free survival dataset, and NB-OS and MM-OS in the overall survival dataset (**Table 1**). These three clinical datasets were studied in the MAQC-II project led by the FDA [19]. To emulate a real-world clinical scenario in applying genomic signatures, two independent populations of patients for each of the three clinical datasets were defined by the MAQC Consortium as the training and validation sets using a chronological approach where the samples in the validation sets were generated at a later date than those in the training sets. The sample sizes for the training sets varied between 130 and 340, and the ratio of positive events to negative events ranged from 0.18 to 1.60; meanwhile, the sample sizes in the validation sets ranged from 100 to 214, and the ratio of positive events to negative events varied between 0.14 and 1.56.

Two positive and two negative control endpoints were also used in this study. The positive control endpoints, i.e., NB-PC and MM-PC, were derived from the NB and MM datasets separately, with the samples denoted by the gender. For the two negative control endpoints, i.e., NB-NC and MM-NC (which correspond to the NB and MM datasets, respectively), the sample labels (i.e., positive or negative events) were randomly generated. Using these two controls allow us to assess the performance of the clinically relevant endpoints against the expected maximum and minimum performance provided by the controls.

### Clinical confidence

The clinical confidence measures the confidence of a sample being assigned to a specific class by a predictive model. For sample *i*, its clinical confidence metric (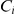) is the confidence level of a sample in being correctly assigned by a predictive model and is defined as:
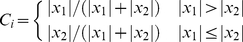
(1)where 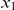 and 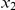 are the similarity measures between sample 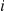 and samples in class 1 and class 2, respectively. The similarity measure varies according to classifiers used. Two well-studied classifiers for gene expression data were employed in this study, i.e., Nearest-Centroid classification rule (*NC*) [4] and *k*-nearest neighbors (*kNN*, *k* = 5) [23]. For the *NC* classifier, 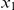 and 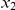 were defined as the correlation coefficients of the unknown sample to the centroids of class 1 and class 2, respectively. The centroid is defined as vectors of the average expression values. For the *kNN* classifier, 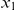 and 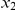 are defined to be the number of nearest neighbors to the unknown sample belonging to class 1 and class 2, respectively.


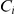
 values range from 0.5 and 1 in which a value of 0.5 indicates that the prediction is due to chance. Increasingly larger 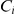 values correspond to increasingly higher prediction confidence. For the sake of simplicity, all of the analysis was based on three confidence levels: low confidence (LC; 0.5≤
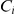
≤0.6), medium confidence (MC; 0.6<
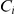
≤0.8) and high confidence (HC; 0.8<
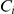
≤1.0).

### Statistical analysis

**Figure 1:**
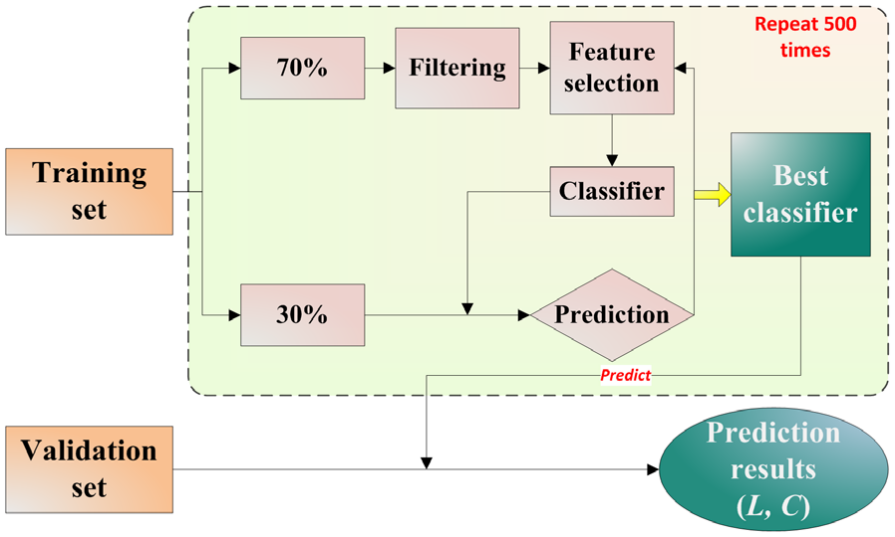
Detailed workflow for correlation analysis of clinical confidence and model performance. Additional details are provided in **Methods S1**.

The general analysis workflow is depicted in **Figure 1**; additional details are provided in **Methods S1**. The analysis protocol starts by developing a best classifier based on the training set, and ends by predicting the validation set. The predicted class and corresponding clinical confidences are recorded in matrices *L* and *C*, respectively. To ensure the statistical validity, the procedure is repeated 500 times, resulting in 500 different classifiers from the training sets and 500 predictions for the validation set. The performance of both training models and predictions is assessed using Matthews correlation coefficient (MCC) [24], [25].

A permutation test was also employed to compare classifier prediction accuracy versus chance [26], [27]. In each permutation, the analysis protocol shown in **Figure 1** was repeated with the exception that the class labels in the training set were randomized. In other words, models constructed with randomized training sets were utilized to predict the validation sets. After 500 repetitions, the degree of chance correlation and predictability of endpoints was computed with Cohen's *d* [28], which measures the standardized difference between two means.

## Results

The cross-validation performance measured in MCC values for all the training models along with the average prediction performance on the validation sets are summarized in **Table S1**. The model performance follows the order of NB-PC, MM-PC, BR-erpos, NB-EFS, NB-OS, BR-pCR, MM-EFS, MM-OS, MM-NC, and NB-NC. The two positive controls performed best while the two negative controls perform worst, which is consistent with expectations from the experiment design.

### Clinical confidence positively correlates with the model prediction performance

**Figure 2:**
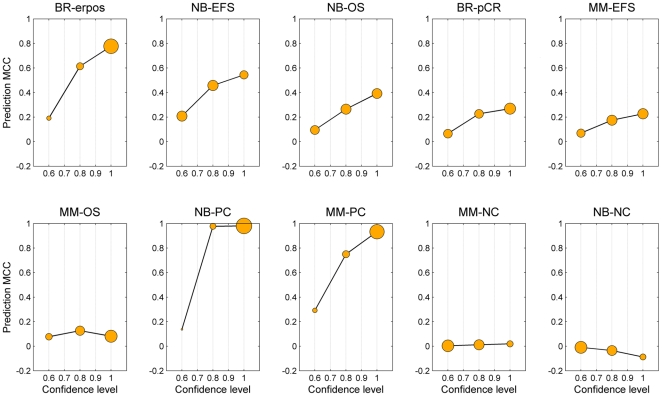
Prediction MCC as a function of clinical confidence for ten datasets using *kNN*. The Circle radii are scaled to the percentage of total samples in the clinical confidence level. The confidence levels are ‘0.6’, ‘0.8’, and ‘1’.

We first investigated the model performance on the validation set for patients falling into different categories of clinical confidence. As depicted in **Figure 2**, a positive correlation is shown between the prediction accuracy and the confidence level for the six clinical and four control endpoints using the *kNN* classifier. Among the six clinical endpoints, the BR-erpos dataset showed the strongest correlation. For the BR-erpos, the average MCC value predictions with low confidence (LC) was only 0.19, while the average MCC value markedly increased to approximately 0.78 as the confidence level approached 1. Thus, compared to the overall MCC value (0.71) (**Table S1**), clinical confidence could successfully account for inter-individual variability in discriminating patients with lower or higher than average prediction accuracy.

**Figure 3:**
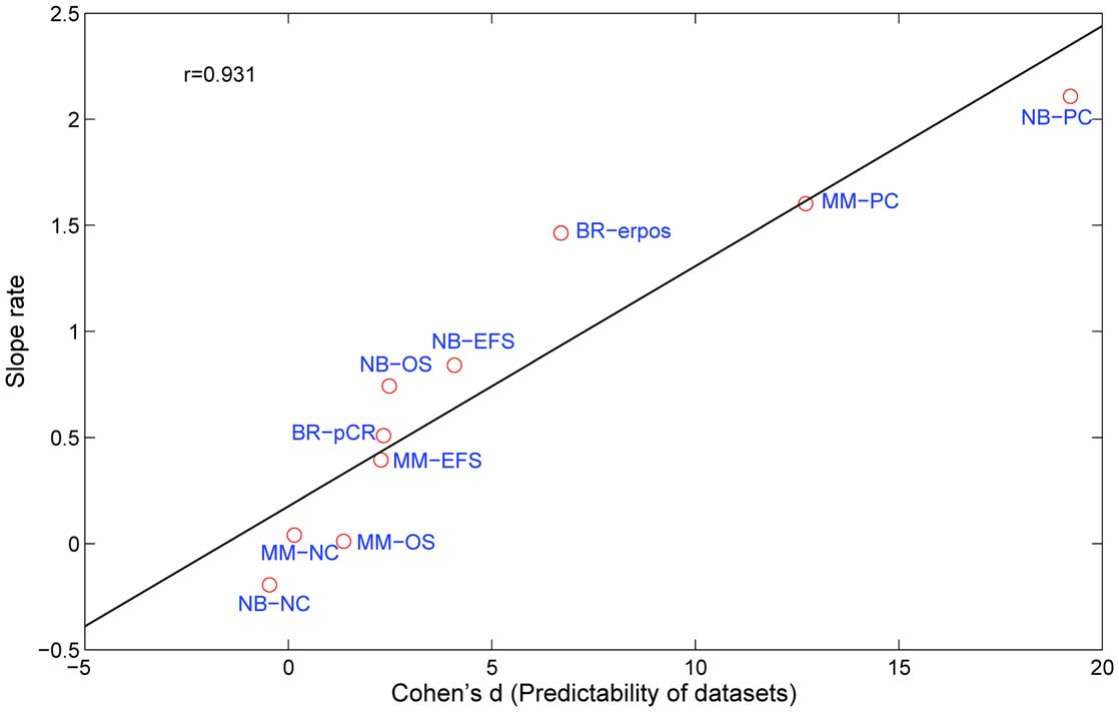
Correlation between slope rate and Cohen's *d* for the *kNN* classifier. The slopes are obtained from regression analysis based on the linear portion of the confidence-MCC curve, while Cohen's *d* represents the inherent predictability of the dataset.

It is clear that the intrinsic predictability by gene expression profiles varies for different endpoints, as evidenced by the gradual decrease in the steepness of model performance for six clinical endpoints over different confidence intervals (i.e., the slopes in **Figure 2**, data was shown in **Table S2**) and the number of samples distributed across different confidence regions (i.e., the marker size in **Figure 2**). As shown in **Figure 3**, a positive linear correlation was observed between the slope obtained from **Figure 2** and the inherent predictability (quantified by Cohen's *d* [28]) of the six clinical and four control endpoints. The predictable endpoints (e.g., BR-erpos, NB-EFS) tended to have a larger percentage of patients (represented as the marker size in **Figure 2**) in the high confidence regions with high prediction accuracy than the less predictable endpoints (e.g., MM-EFS, MM-OS). Detailed information about sample distribution in each confidence region was given in **Table S3**. These observations were further verified using a different pattern recognition method (i.e., *NC*) (**Figures S1 and S2**), and also a different sample splitting strategy (80/20 splitting, **Figures S4 and S5**).

The results demonstrate that a higher inherent predictability of an endpoint is related to a higher percentage of patients that fall into higher confidence levels when using microarray-based predictive models. As the correlation of a genomic signature with a clinical outcome is rarely perfect, the clinical confidence could be useful to separate the patients into different groups for whom specific treatment procedures can be developed.

### The relationship of clinical confidence with patient's survival tim*e*

**Figure 4:**
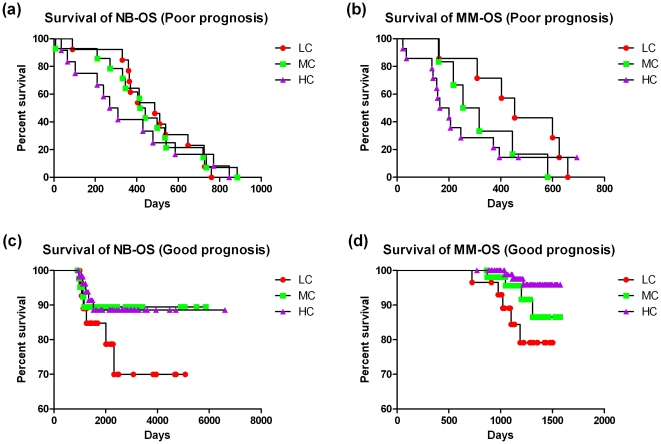
Overall survival (OS) curves for patients with different clinical confidences using *kNN*, where ‘LC’, ‘MC’, and ‘HC’ denote ‘low confidence (0.6)’, ‘medium confidence (0.8)’, and ‘high confidence (1)’, respectively.

We also evaluated whether clinical confidence is predictive of the survival rate for the patients in the validation set. The patients were divided into two prognosis groups (i.e., good and poor prognosis) for both NB and MM datasets with endpoints OS (overall survival) and EFS (event-free survival), respectively (**Methods S1**). **Figure 4** presents the OS curves for patients with different clinical confidences for both prognosis groups. Patients with high clinical confidence exhibited an increased survival rate in the good prognosis group and a decreased survival rate in the poor prognosis group, indicating that the clinical confidence enhanced the accuracy of prognosis derived from the predictive models. Taking MM-OS as an example, the survival rate is apparently higher for patients in the good prognosis group with high confidence (HC) compared to those with low (LC) (log-rank test p value<0.01) and medium (MC) ones (log-rank test p value 0.13), especially for each day mark more than 1000 days (**Figure 4**). For patients with poor prognosis, more than 80% of those with low clinical confidence lived as long as 300 days, while approximately 30% of patients for those with high confidences survived at that time, respectively. Similar trends were also observed in the NB-OS dataset.

**Figure S3**, depicting the EFS curves for patients with different clinical confidences, demonstrates a similar trend as the OS curves presented in **Figure 4**. The positive correlation of clinical confidence with EFS rate is clearly shown in **Figures S3c and S3d** for the patients with good prognosis. However, the correlation is less significant for patients with poor prognosis (**Figures S3a and S3b**). Corresponding results for 80/20 splitting was shown in **Figures S6 and S7**, and conformed to those above-mentioned observations.

The results demonstrate that once the patients were grouped into either good or poor prognosis groups by the predictive models, the clinical confidence can further characterize the survival rate of individual patients in each prognosis group.

## Discussion

Several population-wide diagnostic/prognostic tests based on gene expression have been reported [4], [6], [7]. The population-based models provide only an average indication for the population with corresponding average population accuracy. In this study, we demonstrated that clinical confidence is both capable of separating patients that can be more reliably predicted from those that are less accurately predicted, and predictive of the survival rate for the patients after they are grouped into different prognostic groups. Thus, ascribing a confidence level to prognosis with the clinical confidence metric will provide the clinician a more personalized, objective basis for decisions when using biomarkers derived from microarray data.

Specifically, we found that the clinical confidence provided a better estimation for the survival time when patients were classified into different prognosis categories based on both 70/30 and 80/20 sample assignments. For patients with good prognosis, higher clinical confidence was strongly correlated with longer survival time. Similarly, for patients with poor prognosis, the survival rate was significantly lower for those with high confidences than for the others. Taking endpoints MM-EFS and MM-OS as examples, despite the fact that they are rather difficult to be predicted, patients with high confidence display a significantly higher or lower survival rate when they are grouped in accordance with good or poor prognosis, respectively. Importantly, all patients in the high confidence group survived to 5000 days (**Figure S3c**), demonstrating that clinical confidence is an informative survival time prognosis tool.

An important aspect of this study is that two positive (NB-PC, MM-PC) and two negative control (NB-NC, MM-NC) datasets were involved, which is essential to assess the performance of the clinically relevant endpoints against the theoretical maximum and minimum performance provided by the controls. Specifically, the positive correlation between model performance and clinical confidence for the two positive control datasets shown in **Figure 2** confirmed the potential of clinical confidence to provide a measure of reliability for personalized medicine, while the negligible impact of clinical confidence in the two negative control datasets further limited the possibility of obtaining false positives. Thus, the inclusion of positive and negative control datasets in such an analysis would be of great help to ensure the reliability of the results.

It remains enigmatic why some of the endpoints were more difficult than others to predict. **Figure 2** and **Figure 3** compare predictability across the three datasets and corresponding six endpoints. Readily predictable endpoints have a high percentage of patients who fall into the high confidence region. For example, the percentage of patients that showed high clinical confidence (74.70%) for the BR-erpos endpoint is much higher than that of the MM-EFS endpoint (37.51%) (**Figure 2**), which may indicate that the BR-erpos endpoint contains a stronger gene expression signal than MM-EFS does. Additionally, the predictability of the dataset (Cohen's *d*) is directly related with the correlation coefficient between the confidence level and MCC prediction performance (**Figure 3**).

The ability to quantify clinical confidences may greatly enhance clinical decision-making processes based on microarray-based prediction models, especially for personalized treatment options. For example, the models presented here could test for potential treatment response with the high confidence and low confidence predictions being used in different ways. Patients with good prognosis and high confidences are candidates for applying routine protocols to avoid over-treatment, while rigorous strategies should be selected for those with poor prognosis and high confidences to prolong survival time as long as possible. However, for patients in the low confidence regions, additional evaluation using alternative methods should be considered.

It is important to note that the strategy proposed in this study emphasizing the shift from population-based to personalized cancer prognosis does not negate the importance of population-based prediction, but rather builds upon its success. If the performance of a predictive model is not informative, such as seen in the two negative controls (i.e., MM-NC and NB-NC), the clinical confidence will not be predictive. Thus, model validation methods that include cross-validation and independent external validation are still essential to ensure the validity of microarray-based predictive models. However, since the population-based prediction does not provide an accurate assessment for each patient within the population, clinical confidence offers a means to measure reliability for individual predictions based on the population-based prediction.

The benefits of personalized medicine in health care are well recognized [1]. It allows both the patient and the physician to be more aware of the benefits and risks of possible treatments and potential outcomes affected by genetic make-up or other environmental influences. Thus, informed, tailored, and health-related decisions can be made for each person [29]. Combining microarray technology capable of profiling the expression levels of hundreds of thousands of genes with pattern recognition techniques has been an important step toward individualized decision-making [30]. We presented examples applying confidence assessment to cancer prognosis and survival time prediction for models developed from microarray data. However, the approach can be generalized to biomarkers and models built based on data from other high throughput platforms. Moreover, the concept is generally applicable for all supervised learning classification methodologies that can define a clinical confidence.

## Supporting Information

Figure S1 **Prediction MCC as a function of clinical confidence for ten datasets using** ***NC*** **.** Circle radii are scaled to the percentage of total samples in the clinical confidence level. The confidence levels are ‘0.5–0.6’, ‘0.6–0.8’ and ‘0.8–1’, respectively.
(TIF)

Figure S2 **Correlation between slope rate and Cohen's** ***d*** **for the** ***NC*** **classifier.** The slopes are obtained from regression analysis based on the linear portion of the confidence-MCC curve, while Cohen's *d* represents the inherent predictability of the dataset.
(TIF)

Figure S3 **Event-free survival (EFS) curves for patients with different clinical confidences using** ***kNN*** **where ‘LC’, ‘MC’, and ‘HC’ denote ‘low confidence (0.6)’, ‘medium confidence (0.8)’, and ‘high confidence (1)’, respectively.**
(TIF)

Figure S4 **Prediction MCC as a function of clinical confidence for ten datasets using 80/20 splitting and** ***kNN*** **.** The Circle radii are scaled to the percentage of total samples in the clinical confidence level. The confidence levels are ‘0.6’, ‘0.8’, and ‘1’.
(TIF)

Figure S5 **Correlation between slope rate and Cohen's** ***d*** **for the** ***kNN*** **classifier based on 80/20 sample assignment.** The slopes are obtained from regression analysis based on the linear portion of the confidence-MCC curve, while Cohen's *d* represents the inherent predictability of the dataset.
(TIF)

Figure S6 **Overall survival (OS) curves for patients with different clinical confidences using 80/20 splitting and** ***kNN*** **, where ‘LC’, ‘MC’, and ‘HC’ denote ‘low confidence (0.6)’, ‘medium confidence (0.8)’, and ‘high confidence (1)’, respectively.**
(TIF)

Figure S7 **Event-free survival (EFS) curves for patients with different clinical confidences using 80/20 splitting and** ***kNN*** **, where ‘LC’, ‘MC’, and ‘HC’ denote ‘low confidence (0.6)’, ‘medium confidence (0.8)’, and ‘high confidence (1)’, respectively.**
(TIF)

Table S1 MCC performance for training and validation sets.
(DOCX)

Table S2 Slope and Cohen's *d* for each dataset.
(DOCX)

Table S3 Percentage of patients in low confidence (LC), medium confidence (MC) and high confidence (HC) regions.
(DOCX)

Methods S1 Construction of the best classifier and calculate the correlation between clinical confidence and survival rate.
(DOC)
