# Supplementary figures and images for: Shifting from Population-wide to Personalized Cancer Prognosis with Microarrays
Source: PLoS One. 2012 Jan 25;7(1):e29534. doi: 10.1371/journal.pone.0029534 (PMC3266237; doi:10.1371/journal.pone.0029534)

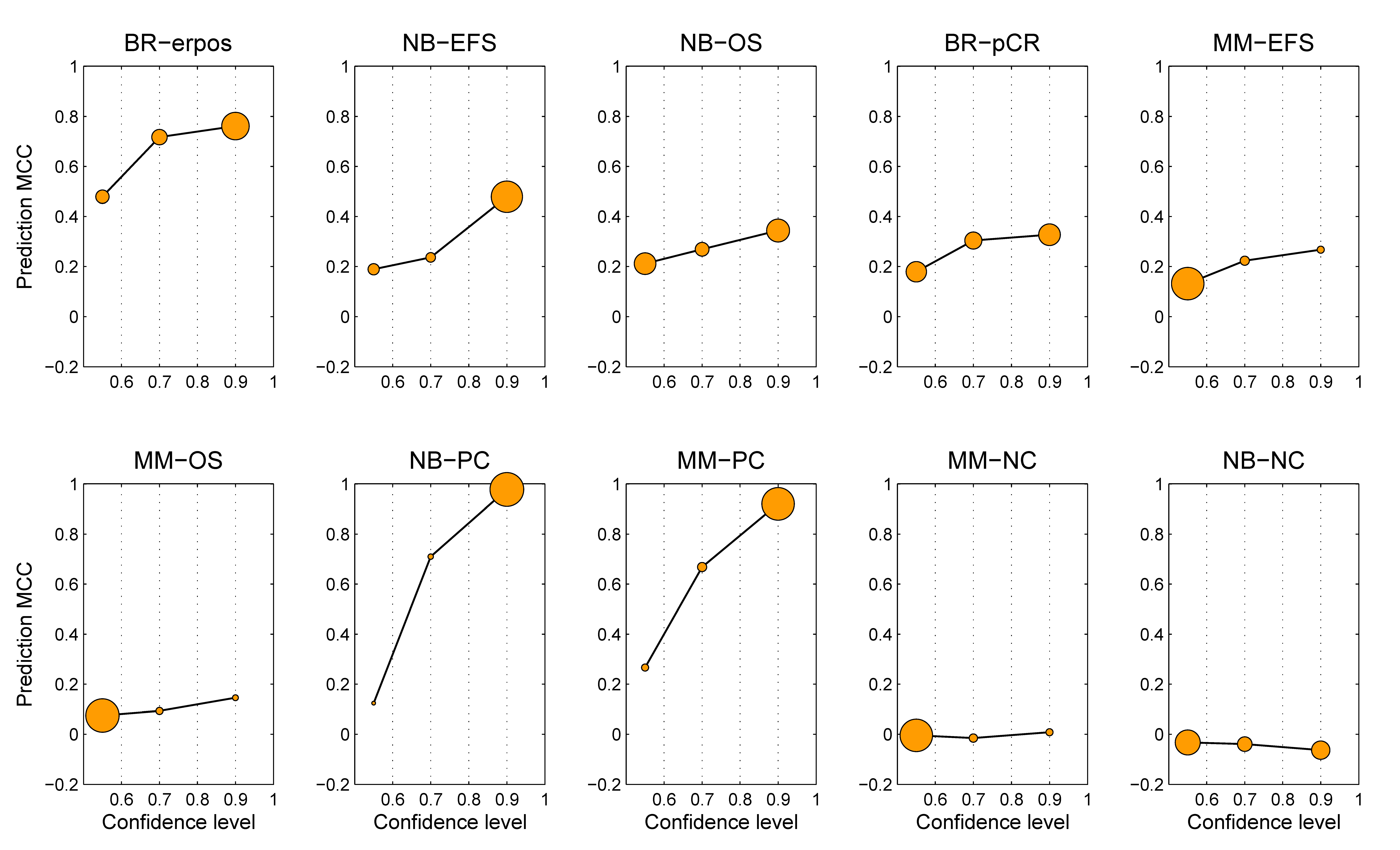

Supplement: Figure S1 — Prediction MCC as a function of clinical confidence for ten datasets using NC . Circle radii are scaled to the percentage of total samples in the clinical confidence level. The confidence levels are ‘0.5–0.6’, ‘0.6–0.8’ and ‘0.8–1’, respectively. (TIF) [file pone.0029534.s001.tif]

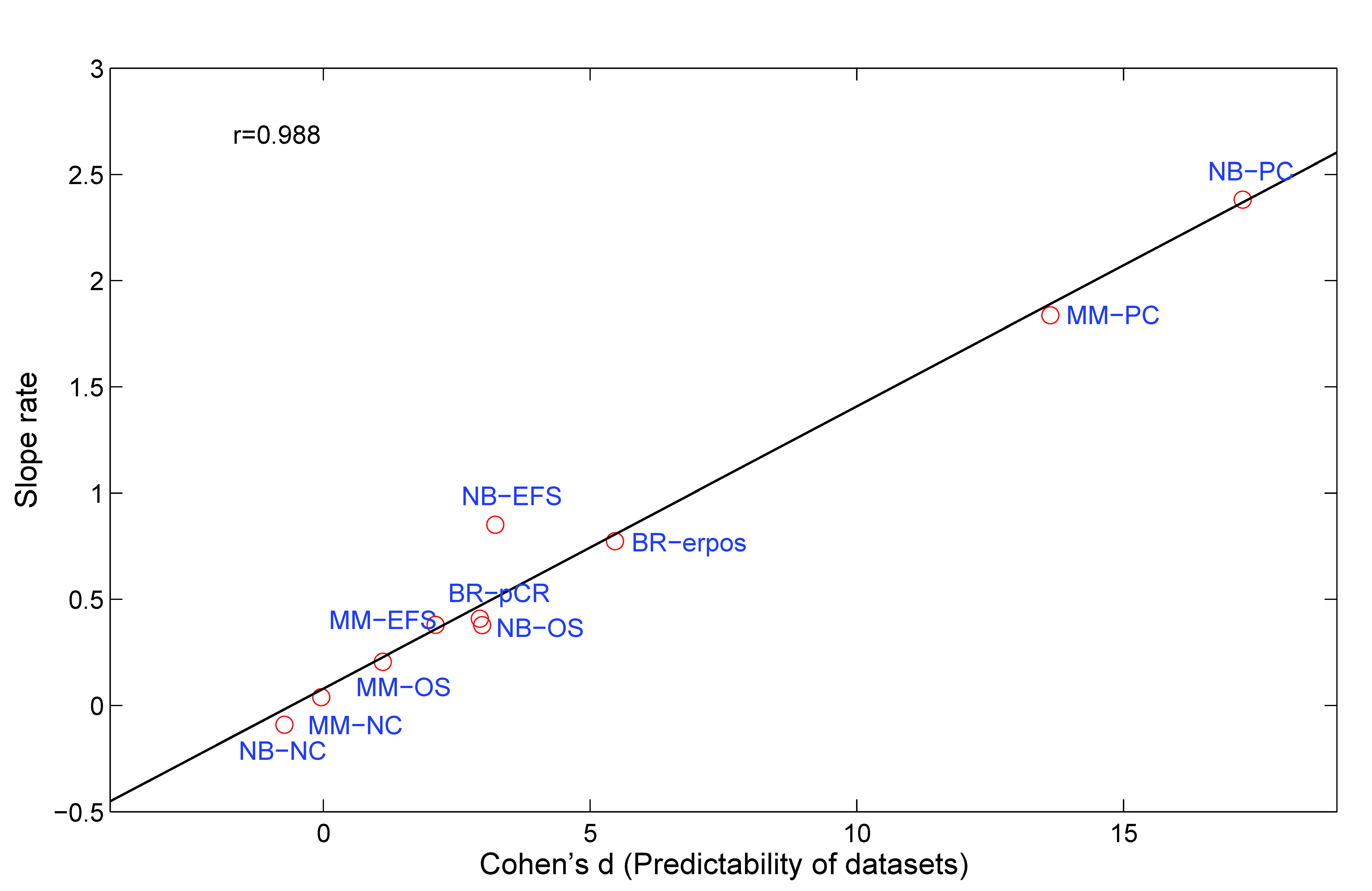

Supplement: Figure S2 — Correlation between slope rate and Cohen's d for the NC classifier. The slopes are obtained from regression analysis based on the linear portion of the confidence-MCC curve, while Cohen's d represents the inherent predictability of the dataset. (TIF) [file pone.0029534.s002.tif]

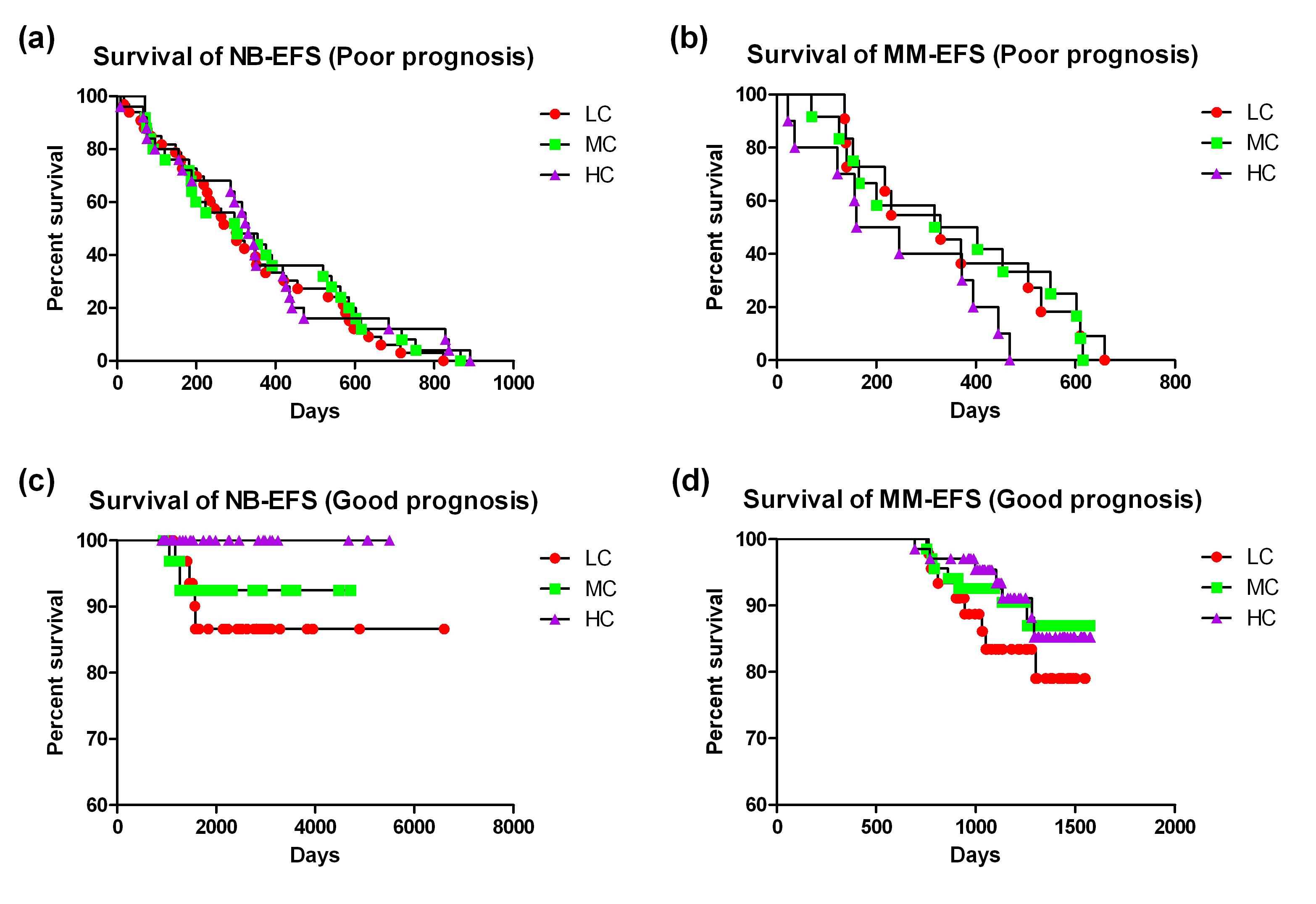

Supplement: Figure S3 — Event-free survival (EFS) curves for patients with different clinical confidences using kNN where ‘LC’, ‘MC’, and ‘HC’ denote ‘low confidence (0.6)’, ‘medium confidence (0.8)’, and ‘high confidence (1)’, respectively. (TIF) [file pone.0029534.s003.tif]

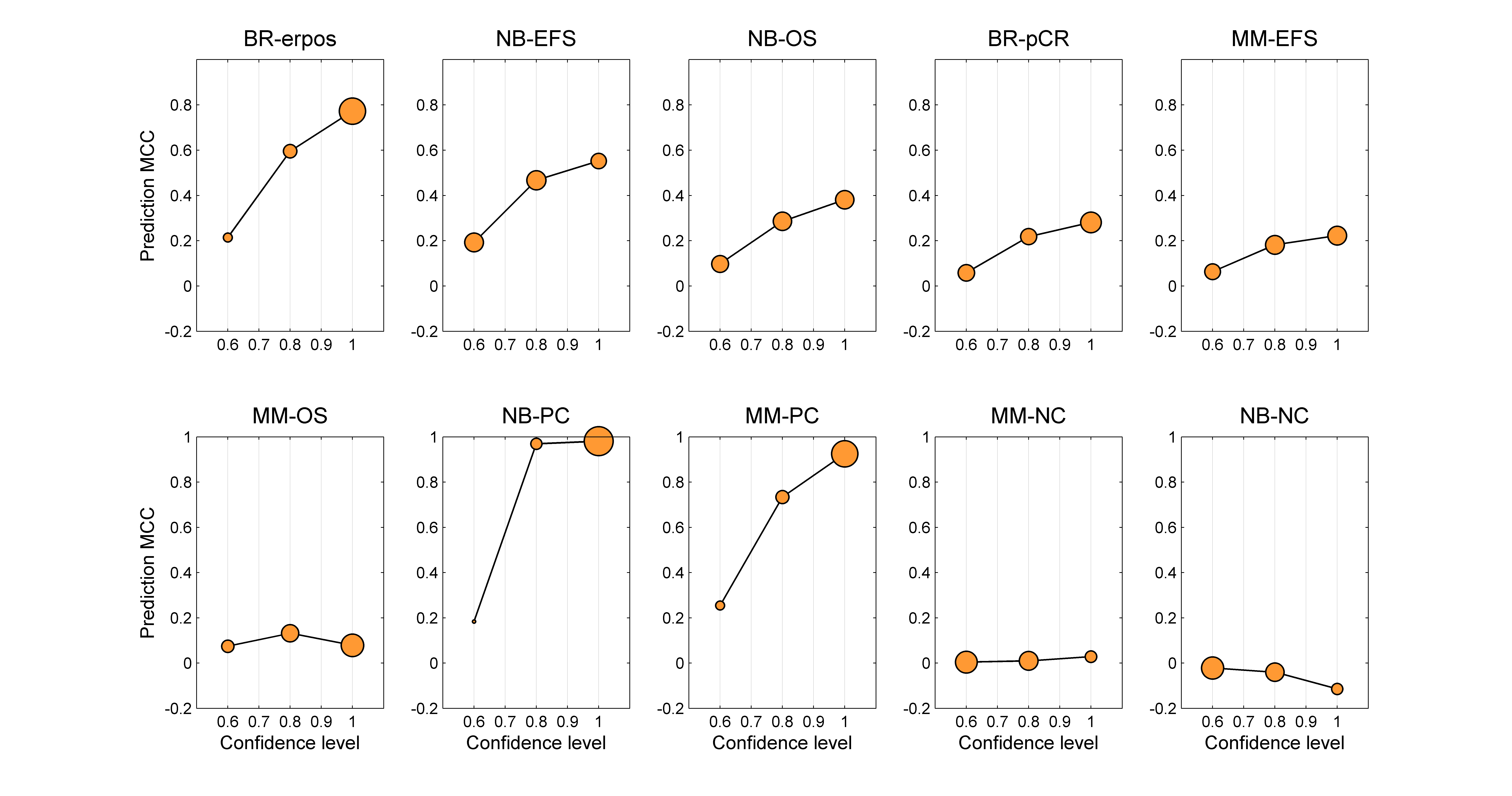

Supplement: Figure S4 — Prediction MCC as a function of clinical confidence for ten datasets using 80/20 splitting and kNN . The Circle radii are scaled to the percentage of total samples in the clinical confidence level. The confidence levels are ‘0.6’, ‘0.8’, and ‘1’. (TIF) [file pone.0029534.s004.tif]

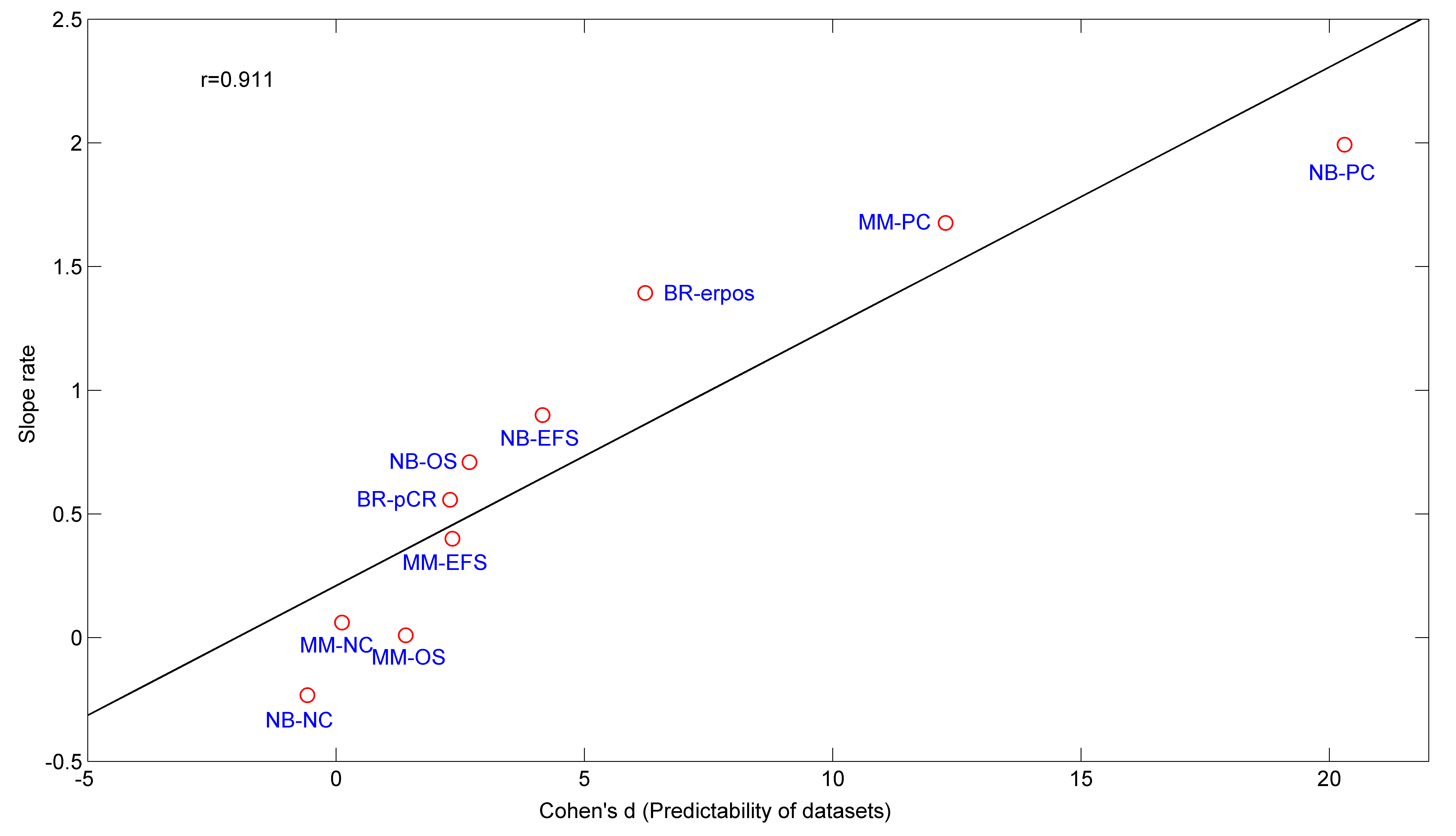

Supplement: Figure S5 — Correlation between slope rate and Cohen's d for the kNN classifier based on 80/20 sample assignment. The slopes are obtained from regression analysis based on the linear portion of the confidence-MCC curve, while Cohen's d represents the inherent predictability of the dataset. (TIF) [file pone.0029534.s005.tif]

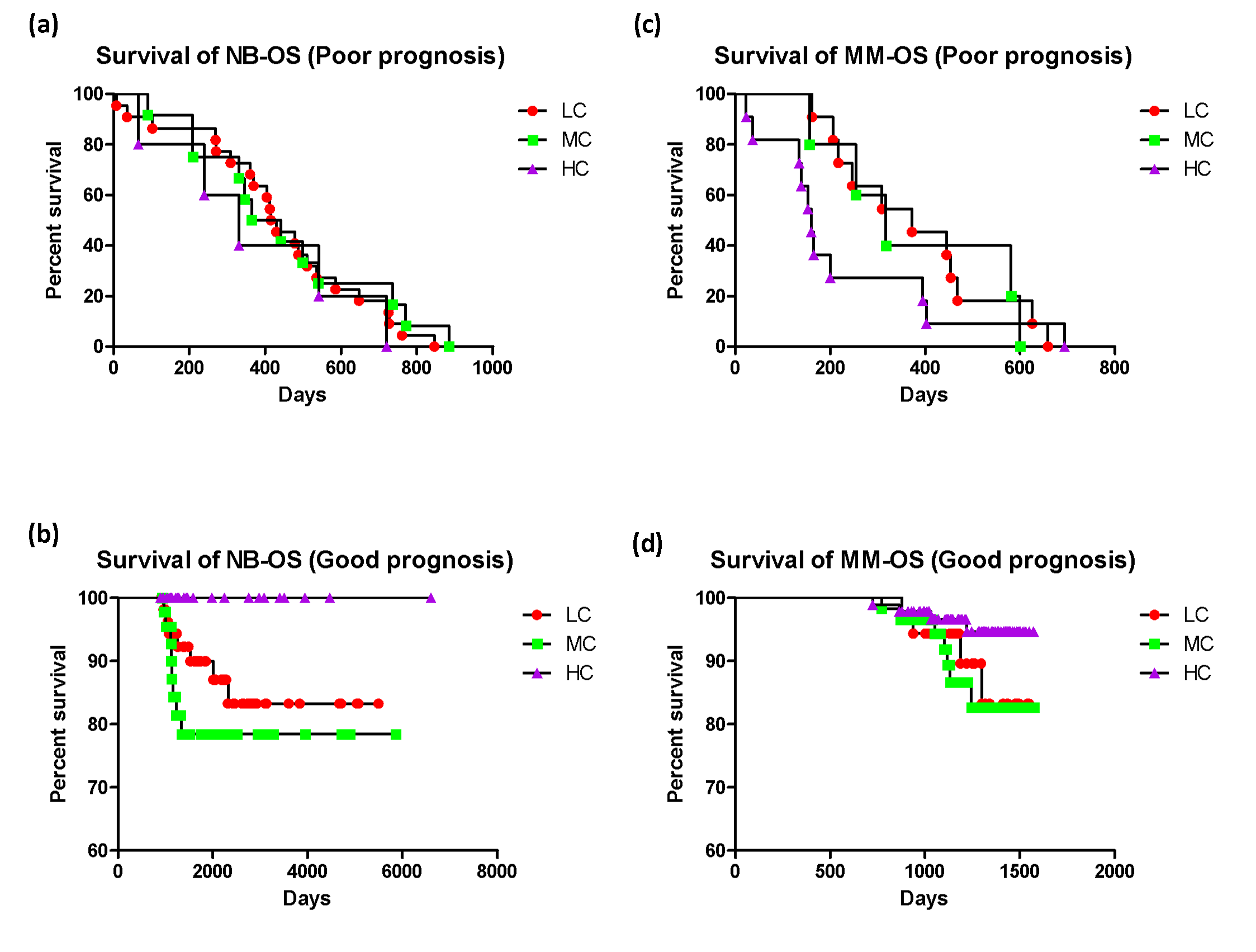

Supplement: Figure S6 — Overall survival (OS) curves for patients with different clinical confidences using 80/20 splitting and kNN , where ‘LC’, ‘MC’, and ‘HC’ denote ‘low confidence (0.6)’, ‘medium confidence (0.8)’, and ‘high confidence (1)’, respectively. (TIF) [file pone.0029534.s006.tif]

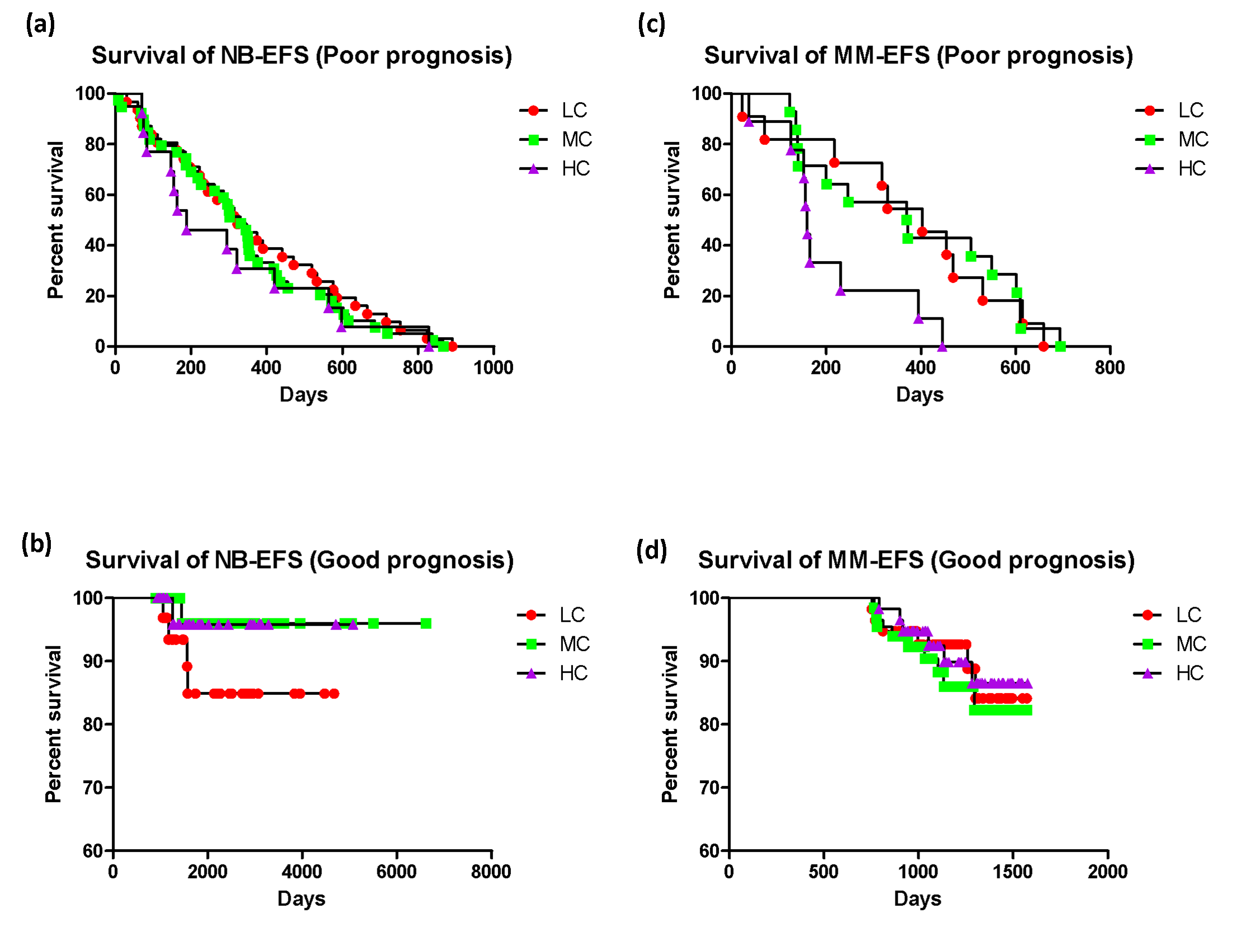

Supplement: Figure S7 — Event-free survival (EFS) curves for patients with different clinical confidences using 80/20 splitting and kNN , where ‘LC’, ‘MC’, and ‘HC’ denote ‘low confidence (0.6)’, ‘medium confidence (0.8)’, and ‘high confidence (1)’, respectively. (TIF) [file pone.0029534.s007.tif]
