# Supplementary material for: Shifting from Population-wide to Personalized Cancer Prognosis with Microarrays
Source: PLoS One. 2012 Jan 25;7(1):e29534. doi: 10.1371/journal.pone.0029534 (PMC3266237; doi:10.1371/journal.pone.0029534)
Supplement: Table S1 — MCC performance for training and validation sets. (DOCX) [file pone.0029534.s008.docx]

**Table S1.** MCC performance for training and validation sets.

|  | *kNN* | | | | | *NC* | | | | |
| --- | --- | --- | --- | --- | --- | --- | --- | --- | --- | --- |
|  | Training* | Validation | | | | Training* | Validation | | | |
|  |  | Overall | LC | MC | HC |  | Overall | LC | MC | HC |
| BR-erpos | 0.86±0.05 | 0.71±0.06 | 0.19 | 0.61 | 0.78 | 0.66±0.06 | 0.71±0.06 | 0.48 | 0.72 | 0.76 |
| NB-EFS | 0.43±0.05 | 0.39±0.06 | 0.21 | 0.46 | 0.54 | 0.46±0.09 | 0.43±0.08 | 0.19 | 0.24 | 0.48 |
| NB-OS | 0.41±0.07 | 0.26±0.09 | 0.10 | 0.26 | 0.39 | 0.35±0.11 | 0.29±0.09 | 0.21 | 0.27 | 0.34 |
| BR-pCR | 0.51±0.09 | 0.23±0.09 | 0.07 | 0.23 | 0.27 | 0.55±0.09 | 0.27±0.07 | 0.18 | 0.30 | 0.33 |
| MM-EFS | 0.38±0.06 | 0.16±0.06 | 0.07 | 0.18 | 0.23 | 0.35±0.10 | 0.15±0.06 | 0.13 | 0.22 | 0.27 |
| MM-OS | 0.36±0.08 | 0.10±0.08 | 0.08 | 0.13 | 0.08 | 0.27±0.12 | 0.08±0.07 | 0.08 | 0.09 | 0.15 |
| NB-PC | 0.94±0.01 | 0.98±0.02 | 0.14 | 0.98 | 0.98 | 0.92±0.03 | 0.96±0.03 | 0.12 | 0.71 | 0.98 |
| MM-PC | 0.84±0.03 | 0.84±0.05 | 0.29 | 0.75 | 0.93 | 0.85±0.03 | 0.87±0.03 | 0.27 | 0.67 | 0.92 |
| MM-NC | 0.28±0.06 | 0.01±0.07 | 0.00 | 0.01 | 0.02 | 0.18±0.11 | -0.01±0.07 | 0.00 | -0.02 | 0.01 |
| NB-NC | 0.21±0.06 | -0.03±0.06 | -0.01 | -0.04 | -0.09 | 0.18±0.10 | -0.04±0.06 | -0.03 | -0.04 | -0.06 |

*****Leave-one-out cross validation results
