# Supplementary material for: Shifting from Population-wide to Personalized Cancer Prognosis with Microarrays
Source: PLoS One. 2012 Jan 25;7(1):e29534. doi: 10.1371/journal.pone.0029534 (PMC3266237; doi:10.1371/journal.pone.0029534)
Supplement: Table S2 — Slope and Cohen's d for each dataset. (DOCX) [file pone.0029534.s009.docx]

**Table S2.** Slope and Cohen’s *d* for each dataset.

|  | Slope | | Cohen's *d* | |
| --- | --- | --- | --- | --- |
|  | *kNN* | *NC* | *kNN* | *NC* |
| BR-erpos | 1.464 | 0.774 | 6.701 | 5.466 |
| NB-EFS | 0.840 | 0.851 | 4.087 | 3.221 |
| NB-OS | 0.743 | 0.378 | 2.485 | 2.976 |
| BR-pCR | 0.509 | 0.408 | 2.344 | 2.932 |
| MM-EFS | 0.394 | 0.380 | 2.279 | 2.102 |
| MM-OS | 0.011 | 0.206 | 1.359 | 1.113 |
| NB-PC | 2.108 | 2.381 | 19.220 | 17.237 |
| MM-PC | 1.602 | 1.836 | 12.715 | 13.630 |
| NB-NC | 0.040 | 0.039 | 0.151 | -0.041 |
| MM-NC | -0.195 | -0.090 | -0.460 | -0.727 |
