# Supplementary material for: Shifting from Population-wide to Personalized Cancer Prognosis with Microarrays
Source: PLoS One. 2012 Jan 25;7(1):e29534. doi: 10.1371/journal.pone.0029534 (PMC3266237; doi:10.1371/journal.pone.0029534)
Supplement: Table S3 — Percentage of patients in low confidence (LC), medium confidence (MC) and high confidence (HC) regions. (DOCX) [file pone.0029534.s010.docx]

**Table S3.** Percentage of patients in low confidence (LC), medium confidence (MC) and high confidence (HC) regions.

|  | *kNN* | | | *NC* | | |
| --- | --- | --- | --- | --- | --- | --- |
|  | LC | MC | HC | LC | MC | HC |
| BR-erpos | 7.59 | 17.72 | 74.70 | 15.56 | 19.86 | 64.59 |
| NB-EFS | 36.02 | 38.61 | 25.37 | 10.82 | 7.81 | 81.37 |
| NB-OS | 28.51 | 36.26 | 35.23 | 39.89 | 16.12 | 43.99 |
| BR-pCR | 27.85 | 27.27 | 44.88 | 34.73 | 25.03 | 40.24 |
| MM-EFS | 25.15 | 37.34 | 37.51 | 87.90 | 7.47 | 4.64 |
| MM-OS | 15.73 | 31.51 | 52.76 | 92.44 | 4.41 | 3.14 |
| NB-PC | 1.43 | 12.97 | 85.60 | 1.37 | 2.57 | 96.06 |
| MM-PC | 8.13 | 18.45 | 73.42 | 4.21 | 7.09 | 88.70 |
| MM-NC | 49.69 | 36.39 | 13.92 | 89.44 | 6.00 | 4.56 |
| NB-NC | 51.49 | 34.80 | 13.72 | 52.87 | 18.12 | 29.02 |
