# Supplementary material for: Shifting from Population-wide to Personalized Cancer Prognosis with Microarrays
Source: PLoS One. 2012 Jan 25;7(1):e29534. doi: 10.1371/journal.pone.0029534 (PMC3266237; doi:10.1371/journal.pone.0029534)
Supplement: Methods S1 — Construction of the best classifier and calculate the correlation between clinical confidence and survival rate. (DOC) [file pone.0029534.s011.doc]

**Methods S1.** Construction of the best classifier and calculate the correlation between clinical confidence and survival rate

**
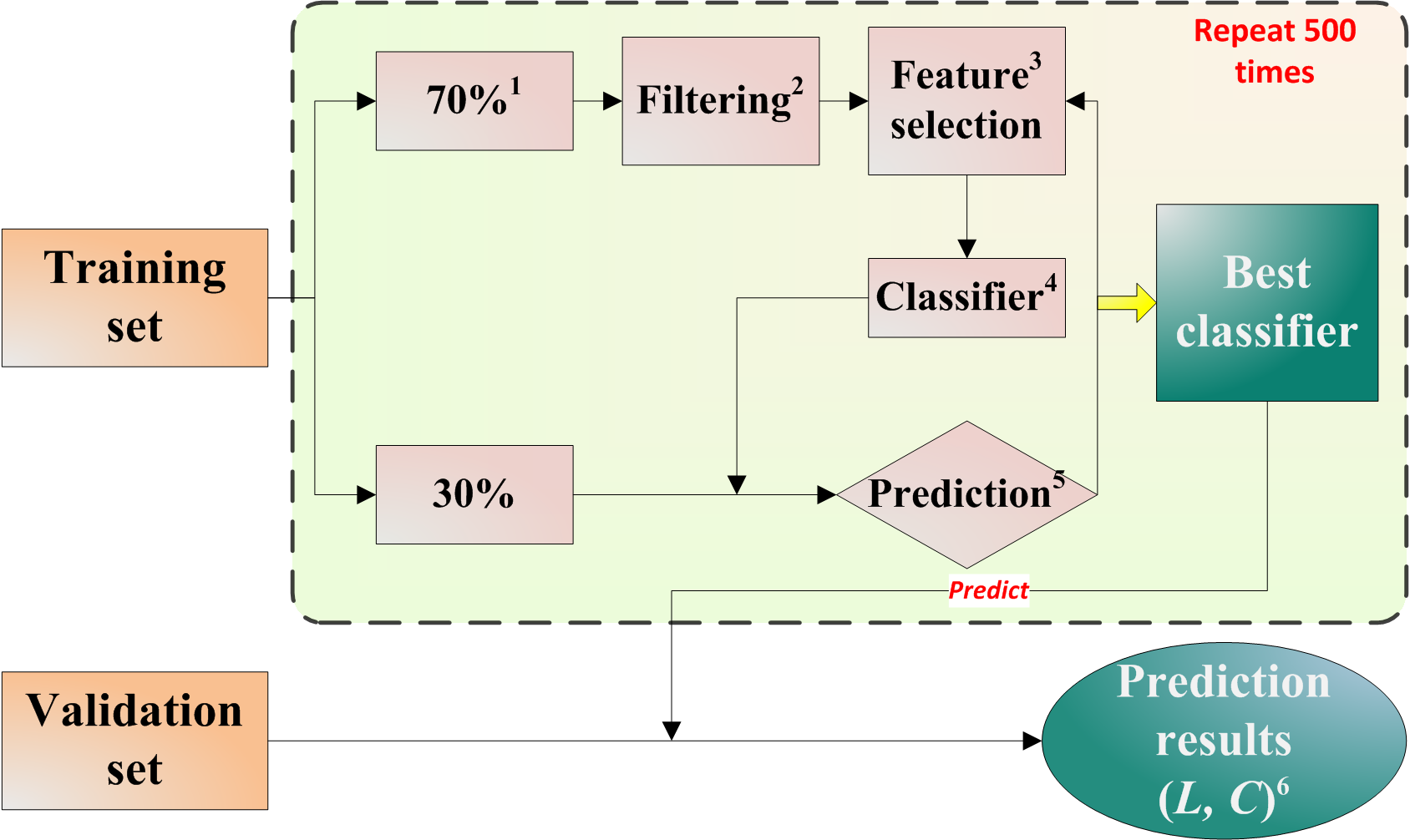
**

In this study, the best classifier was derived from a stratified random sample-splitting (70/30 and 80/20) validation approach. Here 70/30 splitting was utilized to illustrate the main findings in the manuscript, while corresponding results obtained from 80/20 splitting were shown as supplementary materials. Details about classifier construction process are shown as follows using 70/30 splitting as an example (see the superscripts in the figure):

1. Stratified random sample splitting – We used 70/30 splitting, where 70% of the samples are for classifier construction, and the resulting classifier is then used to predict the remaining 30% of the samples to obtain the prediction performance of the classifier. To ensure the statistical validity, we repeated this procedure 500 times, resulting in 500 different classifiers.
2. Filtering – This step is designed to generate an initial pool of probe sets for further analysis. The original pool of probe sets was sorted by the absolute signal-to-noise (SN) ratio, and then the 200 top ranked probe sets were retained for further analysis.
3. Feature selection – We applied a sequential selection method, where the probe set with the highest performance was sequentially added into the model to develop a classifier. The resulting classifier was then used to predict the 30% samples, where the prediction performance was recorded. The process was repeated by incrementally adding one probe set at a time to generate more classifiers.
4. Classifier selection – For classifier *i*, where *i* corresponds to the number of probe sets selected in the classifier, if the performance MCC of next five consecutive classifiers was smaller than or equal to that of classifier *i*, the process was stopped and classifier *i* was selected as the best classifier. Otherwise, Steps 3 and 4 are repeated.
5. Prediction – Base on the best classifier, the predicted labels and corresponding clinical confidences for samples in the validation set were calculated and recorded, resulting in two row vectors*, l* (1×*p*) and *c* (1×*p*), respectively. Here, *p* indicates the number of samples in the validation set.
6. Repetition – Steps 1 to 5 were repeated 500 times, generating two matrices *L*(500×*p*) and *C*(500×*p*), which contained the predicted labels and corresponding confidences, respectively.

To simulate real-world applications as closely as possible, the training and validation sets split by the MAQC Consortium were utilized as two independent patient populations, one for model construction and the other to be prognosticated. Generally, the same protocol shown in the above figure was used again for model construction and prediction, except that no further repetition was needed. Thus, we obtained two vectors *L* and *C* this time, containing the labels and corresponding confidences of the validation patient population, respectively.

Based on the clinical confidence recorded in *C*, we further allocated the patients into different confidence intervals and delineated corresponding survival curves. Considering the significant difference in survival time for patients in different prognosis groups (i.e., good and poor prognosis groups), survival curves with various confidence intervals were delineated for each group using GraphPad Prism 5 (GraphPad Software, Inc.), respectively. It should be noted that the survival data was not used in the classification model, since it serves as independent validation evidence in the study.
